# Impact of the adjacent bone on pseudarthrosis in mandibular reconstruction with fibula free flaps

**DOI:** 10.1186/s13005-023-00389-8

**Published:** 2023-10-03

**Authors:** Claudius Steffen, Ana Prates Soares, Thelma Heintzelmann, Heilwig Fischer, Jan Oliver Voss, Susanne Nahles, Jonas Wüster, Steffen Koerdt, Max Heiland, Carsten Rendenbach

**Affiliations:** 1 Charité – Universitätsmedizin Berlin, corporate member of Freie Universität Berlin and Humboldt-Universität zu Berlin, Department of Oral and Maxillofacial Surgery, Augustenburger Platz 1, 13353 Berlin, Germany; 2 Charité – Universitätsmedizin Berlin, Julius Wolff Institute, Berlin Institute of Health, Augustenburger Platz 1, 13353 Berlin, Germany; 3 Charité – Universitätsmedizin, corporate member of Freie Universität Berlin, Humboldt-Universität zu Berlin and Berlin Institute of Health, Center for Musculoskeletal Surgery, Humboldt-Universität Zu Berlin and Berlin Institute of Health, Augustenburger Platz 1, 13353 Berlin, Germany; 4 Berlin Institute of Health at Charité – Universitätsmedizin Berlin, BIH Biomedical Innovation Academy, BIH Charité Clinician Scientist Program, Charitéplatz 1, 10117 Berlin, Germany

**Keywords:** Mandibular reconstruction, Pseudarthrosis, Cone-beam computed tomography, Fibula, Surgical flaps

## Abstract

**Background:**

Mechanical and morphological factors have both been described to influence the rate of pseudarthrosis in mandibular reconstruction. By minimizing mechanical confounders, the present study aims to evaluate the impact of bone origin at the intersegmental gap on osseous union.

**Methods:**

Patients were screened retrospectively for undergoing multi-segment fibula free flap reconstruction of the mandible including the anterior part of the mandible and osteosynthesis using patient-specific 3D-printed titanium reconstruction plates. Percentage changes in bone volume and width at the bone interface between the fibula/fibula and fibula/mandible at the anterior intersegmental gaps within the same patient were determined using cone-beam computed tomography (CBCT). Additionally, representative samples of the intersegmental zones were assessed histologically and using micro-computed tomography (µCT).

**Results:**

The bone interface (*p* = 0.223) did not significantly impact the change in bone volume at the intersegmental gap. Radiotherapy (*p* < 0.001), time between CBCT scans (*p* = 0.006) and wound healing disorders (*p* = 0.005) were independent risk factors for osseous non-union. Preliminary analysis of the microstructure of the intersegmental bone did not indicate morphological differences between fibula–fibula and fibula–mandible intersegmental bones.

**Conclusions:**

The bone interface at the intersegmental gap in mandibular reconstruction did not influence long-term bone healing significantly. Mechanical and clinical properties seem to be more relevant for surgical success.

## Background

The fibula free flap (FFF) is the gold standard for reconstructing segmental defects of the mandible due to tumors, osteonecrosis and extensive trauma [1, 2]. Postoperative complications like wound healing disorders, plate exposure, fixation failure and incomplete osseous union are common [3]. Especially, pseudarthrosis is regularly observed, the incidence ranging from 24% to more than 45% [3, 4].

Radiotherapy is a known risk factor for pseudarthrosis, but diminished bone healing in mandibular reconstruction is also frequently noted in patients without risk factors [3]. Clinical studies by our group have identified multi-segment reconstructions and the anterior segmental gap as further independent risk factors for pseudarthrosis after mandibular reconstruction, indicating a relevant influence of the mechanics of reconstruction on bone healing at the mandible [3, 5]. The relevance of mechanical strains to stimulate bone healing at the fracture site is also well known from the long bone [6]. Especially, the initial fracture healing phase is sensitive to ideal interfragmentary movements (IOM) [7].

On the other hand, there are studies indicating that the differences between flap and mandible morphophysiology influence the bone healing process after mandibular reconstruction. Yoda et al. showed that cortical bone formed earlier at the osteotomized interface between fibula and fibula compared to the connection interface between fibula and mandibula, thus demonstrating a relevant impact of bone morphology on the development of pseudarthrosis [8]. Similar results indicating better healing between fibula segments have been described by Swendseid et al. [9] and Knitschke et al. [10] However, none of these studies took into account that the analyzed interfaces were in different anatomical regions and therefore exposed to different mechanical loads. Thus, it remains unclear if the mechanics were influencing the development of pseudarthrosis to a higher degree than the morphology of the intersegmental gap.

The aim of this study was therefore to evaluate the impact of morphological factors on bone healing at the intersegmental gap after mandibular reconstruction, by analyzing different bone interfaces (fibula–fibula versus fibula–mandible) at the same anatomical site to minimize mechanical confounders. The hypothesis is that the type and morphology of bone adjacent to the segmental gap does not significantly impact bone healing in the long term.

## Methods

### Patient inclusion criteria and study design

Ethical approval was obtained for this retrospective study (EA2/138/18). The initial patient screening process included all patients who received a FFF for mandibular reconstruction and osteosynthesis using a patient-specific titanium reconstruction plate (2.0 mm, Gebrüder Martin GmbH & Co. KG, Tuttlingen, Germany) at the Department of Oral and Maxillofacial Surgery of Charité – Universitätsmedizin Berlin between August 2017 and April 2022.

Inclusion criteria included the availability of two postoperative cone-beam computed tomography (CBCT) scans. The baseline CBCT scan had to have been performed within the first 30 days after surgery, and the follow-up CBCT scan had to have taken place at least 2 months postoperatively. The minimum interval between the two CBCT scans was 2 months. After 2–3 months, a radiologically detectable callus formation is expected [11]. In order to guarantee bone interfaces between fibula–fibula and fibula–mandible in the same anatomic region within the same patient, exclusively LC-type defects according to Boyd’s classification and a minimum of two fibula segments had to exist [12]. Thus, the bone interfaces exclusively at the anterior region were compared. Fixation of the reconstruction plate was performed using bicortical non-locking screws at the mandible and monocortical non-locking screws at the fibula segments.

Exclusion criteria were secondary reconstructions, major postoperative complications (flap revision or material failure), history of chemo/radiotherapy before the surgery and operative procedures in the anterior region (e.g., plate removal, surgical bone remodeling or refixation) in the time interval between the two CBCT scans.

As described in our previous study, all CBCT scans were performed with the same device (MedSeries H23, Sophisticated Computertomographic Solutions GmbH, Aschaffenburg, Germany) and same adjustments (isotropic voxel edge length of 0.4 mm in all directions), without specific artefact reduction adjustment [5].

For all included patients, gender, age at surgery, surgery date, date of CBCT scans, indication for surgery, number of segments, adjuvant chemo(radio)therapy, diabetes mellitus, nicotine abuse, wound healing disorders, plate exposure and material failure were documented.

### Determination of gap volume and width

**Fig. 1:**
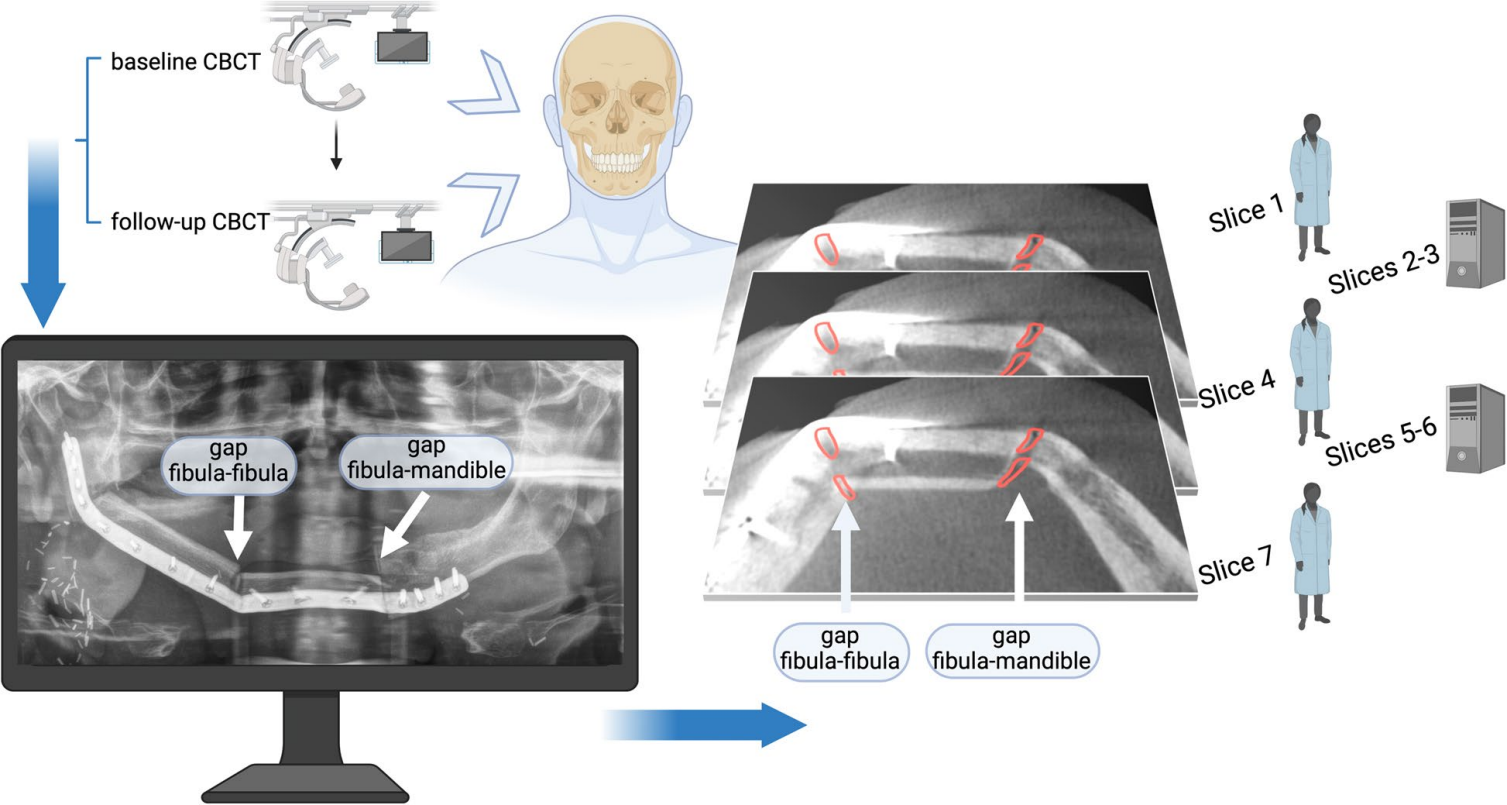
Visualization of the analysis sequence. Two consecutive cone-beam computed tomography scans (CBCTs) are needed for volumetric analysis. Patient inclusion criteria were two anterior segmental gaps including one gap between fibula and fibula bone and another gap between fibula and mandible. DICOM files of CBCTs were imported to the image processing software (Image J). Areas between cortical bones of both intersegmental gaps were identified (red). By analyzing multiple planes of the CBCTs, volume changes were calculated. Every third slice was marked manually. The other slices were interpolated by the software. Figure modified from Steffen et al. [5] Created with Biorender.com

Determination of intersegmental gap volume and width was performed according to our previously described methodology (Fig. 1) [5]. Two DICOM files of CBCT scans were analyzed for each patient using the image processing software Image J (ImageJ for Java 8, version 1.53f, National Institute of Health, Bethesda, Maryland, USA). Each analysis consisted of a first scan shortly after the initial reconstruction and a second scan in the postoperative course. The volume and gap width were exclusively analyzed for the anterior gaps for both the intersegmental gap between the fibula and the native mandible and secondly between the fibula and fibula bone. Images were rotated, so that the area in between the lingual and buccal cortex of each bone interface could be determined. Drawing of the gap volume was performed manually every three slices and otherwise interpolated by the software. The quantification of all slices resulted in the total volume (TV [mm^3^]). Since the initial TV was determined as a baseline value, a reduction of the TV in the follow-up CBCT could be judged as ossification. Therefore, a complete osseous union (100% union) was found in cases with a 100% volume change, and a non-union as a volume change of 0%. Intermediate unions had a volume change between 0 and 100%.

In order to allow a more distinct analysis, separate analyses of both the superior and inferior parts of each gap were performed, including separate determination of the gap width (mm, superior and inferior) in the initial CBCT scan. For the overall comparison of gap widths between both bone interfaces, the mean values of gap widths between the buccal and lingual cortex were used.

### Histological assessment and micro-computed tomography

Exemplary bone samples (2–4 mm) of both anterior intersegmental gaps were harvested during hardware removal from two different patients that showed good osseous healing at the anterior and posterior gaps, using a trephine bur. All samples were fixated in 4% paraformaldehyde for 3 days. The samples from one patient underwent histological processing, and were decalcified, dehydrated and embedded in paraffin. The samples were cut into Sects. (3 µm) and the resulting slides were stained with hematoxylin and eosin (HE) and Picro-Sirius Red staining. Light microscopy was used for the evaluation of the HE slides (LeicaDM6B, Leica Biosystems), while a polarized modus was used to image the Picro-Sirius Red-stained slides.

The samples from the other patient were imaged by means of micro-computed tomography (µCT) at the beamline ANATOMIX of the French national synchrotron facility SOLEIL (Paris, France) [13]. The X-ray beam energy was about 45 keV. A CMOS detector (Hamamatsu Orca Flash 4.0 V2) coupled with a scintillator resulted in an effective pixel size of 0.87 μm. A total of 3600 (fibula–fibula sample) and 4700 (fibula–mandible sample) radiographic projections were recorded (250 ms exposure time per frame) while continuously rotating the samples over 360° in half-acquisition mode, with a sample-to-detector distance of about 50 mm. The volumes were reconstructed using an in-house Python-based reconstruction platform, and a modified Paganini-based filter was used for enhancing contrast (δ/β ratio of 60) [14].

### Statistical analysis

Patient data were collected using Microsoft Excel (Microsoft Corporation, Redmond, USA). Descriptive analysis was used to demonstrate patient characteristics. Differences in volume change and gap width between gap sites (superior/inferior) were analyzed using unpaired t-tests for normally distributed data. Linear regression was used to identify risk factors (independent variables) for volume change (dependent variable). The level of significance was set at 5% (*p* = 0.05) for all analyses. All statistical analyses were performed using SPSS (version 29.0., IBM Corp., Armonk, New York, USA).

## Results

**Fig. 2:**
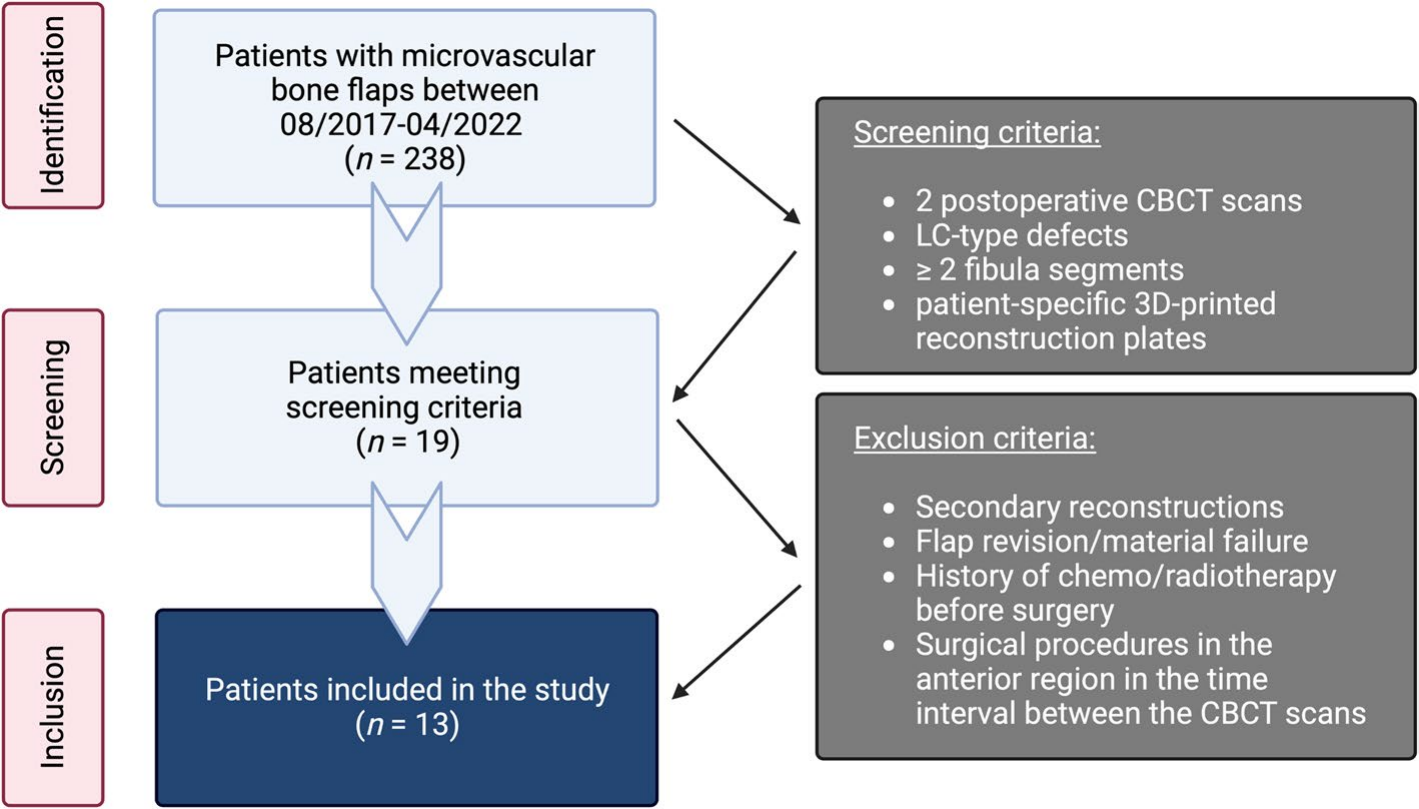
Patient inclusion criteria. Screening criteria were met in 19 patients. Six patients were excluded due to confounders, resulting in 13 patients being included in the present study

The inclusion criteria were met by 13 patients (eight male, five female) (Table 1, Fig. 2). The average time between baseline and follow-up CBCT was 42.0 (± 22.8) weeks. There were no cases of plate exposure or material failure in the patient cohort.

**Table 1:** Patient characteristics

Variable	Category	Mean (± SD) /frequency (%)
Gender	Female	5 (38.5%)
	Male	8 (61.5%)
Age at surgery		59.7 (± 14.5)
Interval between surgery and baseline CBCT (days)		10.0 (± 7.2)
Interval between baseline and follow-up CBCT (weeks)		42.0 (± 22.8)
Indication for surgery	Squamous cell carcinoma	8 (61.5%)
	Osteoradionecrosis	3 (23.1%)
	ARONJ	1 (7.7%)
	Osteomyelitis	1 (7.7%)
Number of segments	2	11 (84.6%)
	3	2 (15.4%)
Adjuvant radiotherapy	Yes	4 (30.8%)
	No	9 (69.2%)
Adjuvant chemotherapy	Yes	0
	No	13 (100%)
Diabetes	Yes	1 (7.7%)
	No	12 (92.3%)
Nicotine abuse	Yes	6 (46.2%)
	No	7 (53.8%)
Wound healing disorder/Fistula	Yes	3 (23.1%)
	No	10 (76.9%)
Plate exposure	Yes	0
	No	13 (100%)
Material failure (plate fracture)	Yes	0
	No	13 (100%)

(*ARONJ* antiresorptive agent-related osteonecrosis of the jaw, *CBCT* cone beam computed tomography)

The volume change was analyzed for each patient for both the mandibular–fibular and fibular–fibular interfaces. Overall, 26 gaps were analyzed and on average 12 slices were analyzed for each gap depending on osseous union and the height of the fibula bone. As indicated in Table 2, univariate analysis showed that the gap interface did not significantly influence volume change and that the interval between CBCT scans (*p* < 0.001), indication for surgery (*p* = 0.008), adjuvant radiotherapy (p < 0.001) and diabetes (*p* = 0.035) significantly impacted osseous union. All variables were forwarded to multivariate regression analysis (Table 3). The interval between CBCT scans (*p* = 0.006), indication for surgery (*p* = 0.017), adjuvant radiotherapy (*p* < 0.001) and wound healing disorder/fistula (*p* = 0.005) were independent risk factors for diminished gap healing. The gap interface (*p* = 0.223) did not significantly influence the volume change of the intersegmental gaps.

**Table 2:** Univariate linear regression analysis with volume change as dependent variable

Variable	Category	Mean volume change (%) (± SD)	*p*-Value, B
Initial gap width			0.256, -9.938
Gap interface	Fibula-Fibula	66.34 (± 28.62)	0.287, 15.438
	Fibula-Mandible	50.91 (± 42.36)	
Gender	Female	75.71 (± 33.60)	0.952, -0.853
	Male	47.95 (± 34.65)	
Age at surgery			0.264, 0.519
Interval between CBCT scans			< 0.001, 7.514
Indication for surgery	SCC	64.91 (± 37.02)	0.008, -42.908
	Osteoradionecrosis	39.95 (± 32.54)	
	MRONJ	87.76 (± 17.30)	
	Osteomyelitis	35.24 (± 35.27)	
Number of segments	2	59.46 (± 36.26)	0.257, -56.304
	3	54.05 (± 41.63)	
Adjuvant radiotherapy	Yes	42.85 (± 39.95)	< 0.001, -117.935
	No	65.64 (± 33.31)	
Diabetes	Yes	19.76(± 19.00)	0.035, -112.422
	No	61.86 (± 35.68)	
Nicotine abuse	Yes	43.17 (± 34.26)	0.184, -28.343
	No	71.87 (± 33.60)	
Wound healing disorder/fistula	Yes	63.98 (± 37.46)	0.677, -13.299
	No	57.02 (± 36.76)	

(*ARONJ* antiresorptive agent-related osteonecrosis of the jaw, *SCC* squamous cell carcinoma, *CBCT* cone-beam computed tomography, *B* regression coefficient)

**Table 3:** Regression analysis of all variables with volume change as the dependent variable

Variable	*p*-Value	95% CI
Initial gap width	0.447	-21.0, 9.8
Gap interface	0.223	-8.4, 33.0
Gender	0.307	-15.1, 44.5
Age at surgery	0.821	-0.8, 1.0
Interval between CBCT scans	0.006	2.3, 11.0
Indication for surgery	0.017	-22.7, -2.6
Number of segments	0.091	-7.1, 85.6
Adjuvant radiotherapy	< 0.001	-114.3, -42.3
Diabetes	0.752	-57.8, 42.7
Nicotine abuse	0.903	-31.8, 35.7
Wound healing disorder/fistula	0.005	-112.3, -24.5

*CBCT* cone-beam computed tomography

Analysis of the complete gap revealed no significant differences in initial gap width or volume change, nor in the comparison of inferior and superior parts of each gap (Table 4). Also, in the regression analysis with volume change as a dependent variable, the gap parts (inferior versus superior) and gap width could not be identified as significant independent variables for either interface (fibula–fibula or fibula–mandible) (Table 4).

**Table 4:** Mean gap widths and volume changes of different gap sites (inferior/superior). Additional linear regression analysis including gap width and gap site (inferior/superior) as independent variables on volume change (dependent variable)

Gap site		Mean initial gap width (mm) (± SD)	Mean volume change (%) (± SD)	Variable gap width in regression analysis with gap site (inferior/superior) (p; 95%-CI)	Variable gap site (inferior/superior) in regression analysis with gap width (p; 95%-CI)
Fibula-Fibula	Total	0.88 (± 0.36)	66.34 (± 28.62)		
Fibula-Mandible	Total	1.44 (± 1.09)	50.91 (± 42.36)		
	t-test	0.090	0.287		
Fibula-Fibula	Inferior	0.78 (± 0.51)	69.92 (± 32.15)		
	superior	0.78 (± 0.60)	52.55 (± 42.48)		
	t-test	0.995	0.251	0.400 (-16.81, 40.57)	0.255 (-13.39, 48.09)
Fibula-Mandible	Inferior	0.93 (± 0.58)	51.25 (± 46.13)		
	Superior	1.21 (± 1.15)	46.64 (± 42.99)		
	t-test	0.446	0.794	0.909 (-19.92, 22.29)	0.787 (-32.47, 42.34)

**Fig. 3:**
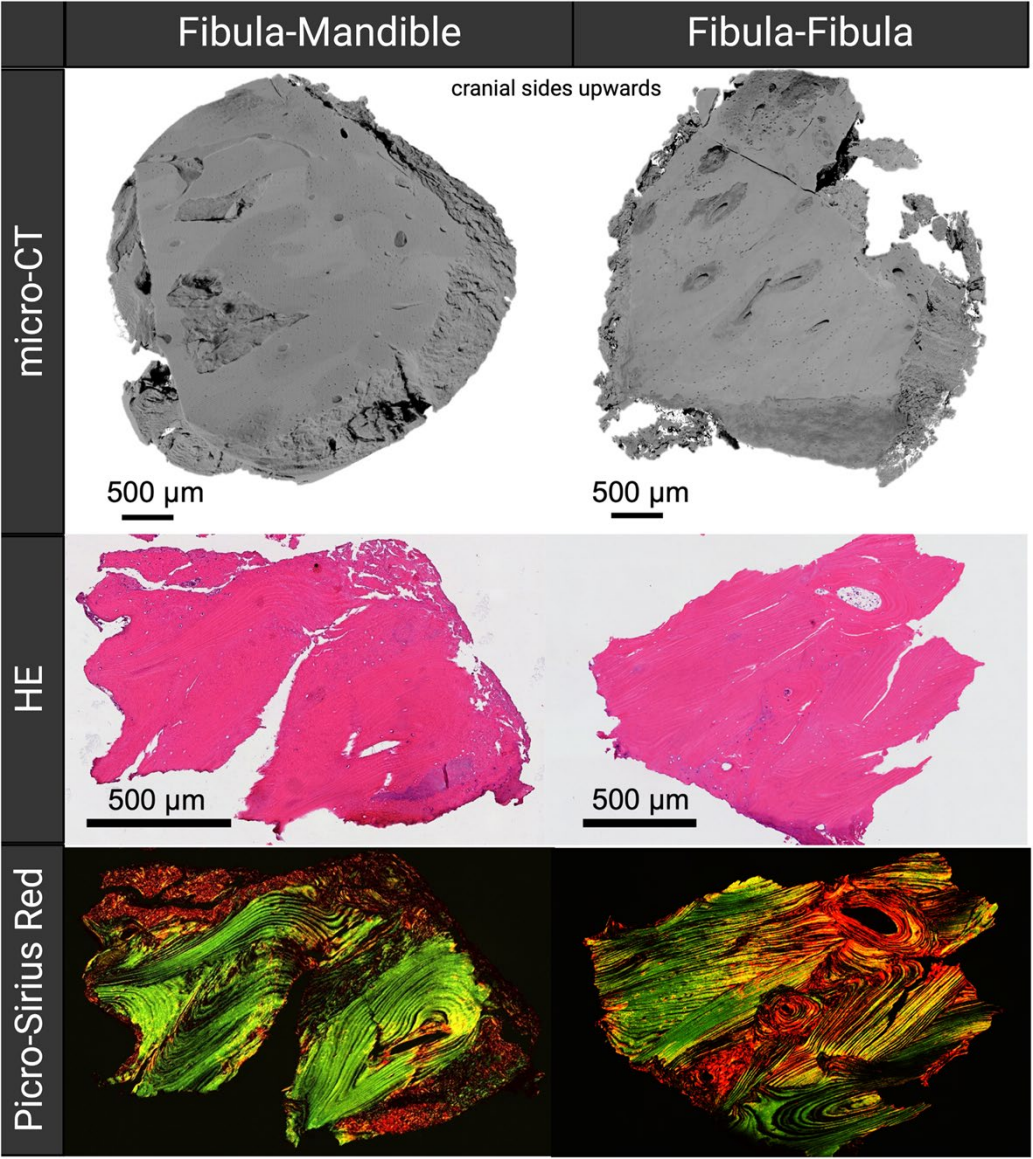
Micro-computed tomography (µCT) of biopsies from both intersegmental gaps. The fibula–mandible intersegmental zone shows a less compact and more heterogenous structure (mixture of dark and bright grey areas) in the µCT. The structure of the fibula–fibula intersegmental bone sample demonstrates similar characteristics as fibula cortical bone with a homogenous structure. The histological observations (HE and Picro-Sirius Red) demonstrated a similar microstructure of both samples from the intersegmental zones. No differences in the shape of type I (yellow–red) or type III (green) collagen fibers were noted. Created with Biorender.com

The preliminary morphological observation of the intersegmental bone samples revealed a more compact and homogenous structure similar to the fibula cortical bone characteristics in the fibula–fibula intersegmental bone, while the mandible–fibula intersegmental bone seemed to have a less compact and more heterogenous structure (Fig. 3). Morphological inspection of the Picro-Sirius Red-stained slides revealed a similar collagen microstructure in both intersegmental bones, although the fibula–fibula slide showed a more organized structure with the fibers circumventing the Haversian canals. The samples were taken from patients 11 months (for µCT) and 13 months (for histology) after reconstructive surgery. Further characteristics of these patients are demonstrated in Table 5.

**Table 5:** Characteristics of patients who underwent bone sample biopsies

Variable	Patient for µCT	Patient for histology
Gender	Male	Female
Age at initial surgery	66	65
Interval plate removal after initial surgery (months)	13	11
Osseous union at plate removal surgery	Very good	Very good
Interval between baseline and follow-up CBCT (weeks)	4	2
Indication for surgery	Osteomyelitis	Squamous cell carcinoma
Number of segments	2	2
Adjuvant radiotherapy	No	No
Adjuvant chemotherapy	No	No
Diabetes	No	No
Nicotine abuse	Yes	Yes
Wound healing disorder/Fistula	No	No
Plate exposure	No	No
Material failure (plate fracture)	No	No

(*CBCT* cone beam computed tomography)

## Discussion

This study aimed to assess if the type of bone adjacent to the intersegmental gap affects long-term osseous healing after mandibular reconstruction with patient-specific 3D-printed titanium reconstruction plates. Volumetric assessment of repetitive CBCT scans, as well as µCT and histological analyses, was used to quantitatively and qualitatively evaluate bone healing. To the authors’ knowledge, this is the first study using this combination of assessments to analyze bone healing at the mandible.

The results of this study show no significant differences in volume change between fibula/fibula and fibula/mandible interfaces. Therefore, there seems to be no relevant impact of the bone adjacent to the intersegmental gap in mandibular reconstruction on the rate of osseous union in the long term. This finding is contrasted by that of the study of Yoda et al., who stated that bone adjacent to the gap influences the bone healing process [8]. In that study, CT data of a 66-year-old patient who received mandibular reconstruction with a FFF were analyzed and a finite element (FE) model was created to quantify mechanobiological responses. Hereby, it was shown that cortical bone formed earlier at the osteotomized interface between fibular and fibular bone. However, there was only one patient included in the study and, more importantly, the bone interfaces were at different anatomical sites, resulting in different mechanics. Disregard of anatomical sites and therefore differing mechanical stimuli were also shortcomings of other studies: Swendseid et al. analyzed maxillary and mandibular defects in 104 flap reconstructions and found a significantly higher rate of complete unions between osteotomized free flap segments compared with flap segments and native bone (65% versus 53%) [9]. However, fibula, scapula and radial forearm flaps as well as maxillary and mandibular defects were combined in the analysis. Also, Knitschke et al. combined maxillary and mandibular defects in the analysis of 133 patients who underwent reconstructions using FFFs [10]. Further shortcomings of both studies were the lack of multivariate analyses concerning the difference in osseous union between different bone interfaces, subjective radiological analyses without the volumetric methodology and the combination of different anatomical regions. Furthermore, the initial gap size was not taken into account, although its importance and confounding influence has been previously demonstrated [5, 15].

The present study overcomes these limitations by using the volumetric assessment of intersegmental bone volumes using repetitive CBCT scans, as previously described [5]. This methodology allows an objective evaluation of radiological pseudarthrosis, which is a relevant improvement compared to the subjective, visual determinations by other studies [3, 15–18]. Furthermore, the present study minimized the effect of mechanical confounders by only including patients who underwent reconstructions using patient-specific 3D-printed titanium reconstruction plates and multi-segment reconstructions with two intersegmental gaps in the anterior region. This allowed analysis of the interfaces between mandible/fibula and fibula/fibula in a similar anatomical region within the same patient. The reduction of mechanical confounders is especially important, because mechanics have been previously described as an important factor in bone healing [19]. In mandibular reconstruction, this has been proven by the relevant influence of different types of osteosynthesis plates [20] and the gap site (anterior versus posterior) [5] on the development of pseudarthrosis. Postoperative non-occlusion as a further mechanical variable did not significantly influence non-union rates in a previous study [3]. In this study, postoperative occlusion was only present in one patient. Therefore, this study cannot analyze the impact of dentition and further studies using adequate bite force measurement devices are needed [21].

**Fig. 4:**
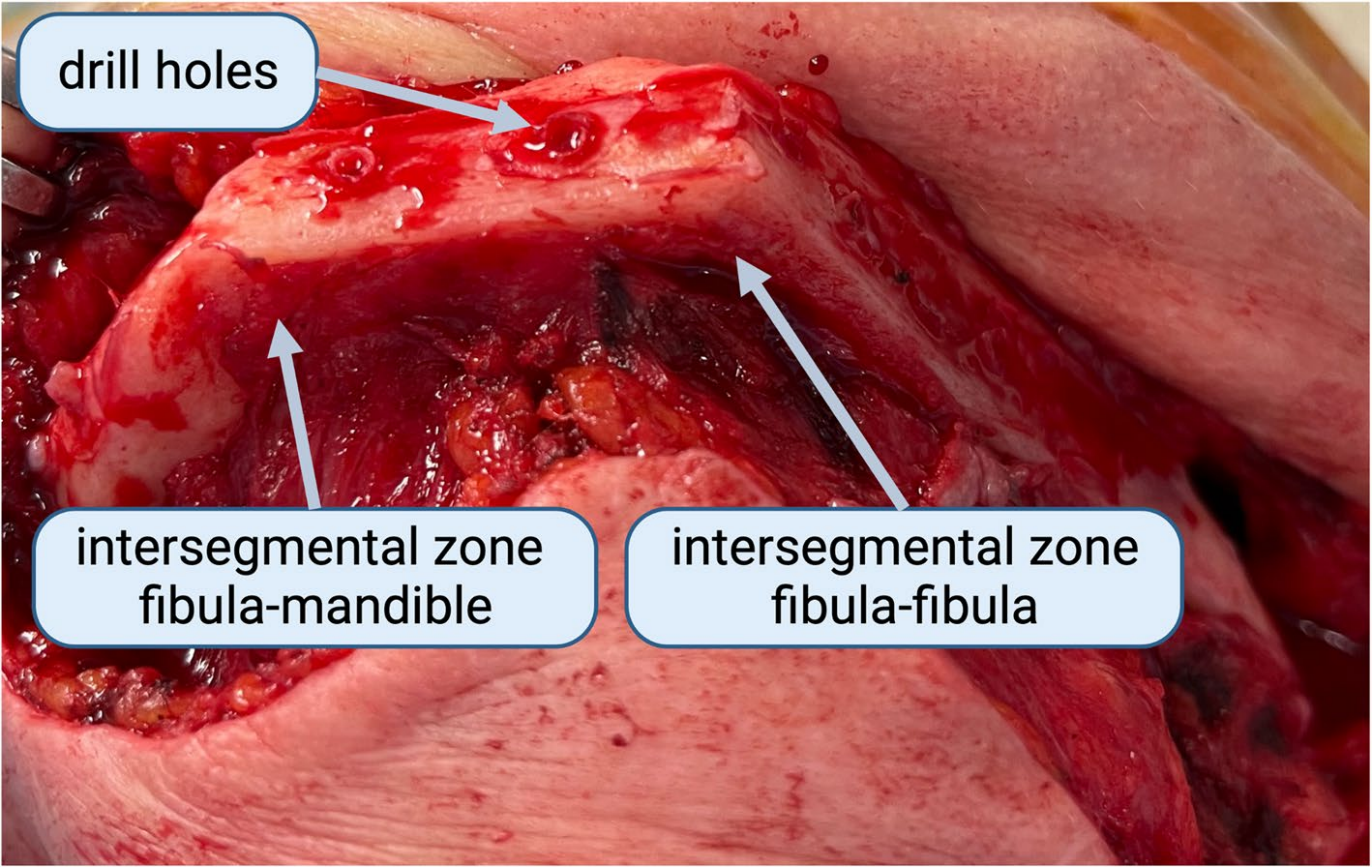
Clinical demonstration of osseous healing after mandibular reconstruction. A 65-year-old female patient underwent plate removal 11 months after having received a two-segment fibula free flap. There was complete osseous union at both anterior intersegmental gaps. Bleeding from the former drill holes after plate removal indicated good vascularization of the flap. Created with Biorender.com

Compared to the previously mentioned studies the use of multivariate analysis is a further strength of the current study. Radiotherapy, which had already been identified as a risk factor for pseudarthrosis [22], was confirmed as an independent risk factor for diminished osseous healing. Also, the relevant influence of time on osseous healing was confirmed by the present study and its influence was taken into account using the regression analysis [10]. The insignificant impact of the initial gap size on osseous healing shown by the multivariate analysis presumably resulted from the limited sample size of the present study. The relevant influence of the indication for surgery and wound healing disorders on osseous healing may also be a result of this and need to be further investigated by studies focusing specifically on these factors. Despite the inclusion of all these factors in the regression analysis, the type of bone adjacent to the gap remained an insignificant factor in the development of pseudarthrosis (Fig. 4).

This finding is remarkable considering that differences between the mandible and other bones are clinically apparent. For example, cherubism and antiresorptive agent-related osteonecrosis of the jaw (ARONJ) are both pathognomonic for the jaw bone [23, 24]. In order to reveal the specifics of the mandibular bone compared to other bones, morphogenetical and morphological analyses were the focus of previous studies: The mandible originates from neural crest mesenchyme (NCM), while the fibula bone has a mesodermic origin [25]. Findings of greater osteogenic potential of the mandible compared to other skeletal bones have been previously described and might be related to the difference in morphogenesis [24, 26]. Mandible bone marrow stromal cells formed 70% larger bone nodules with a three-fold more mineralized bone after implantation into nude mice, in comparison to cells derived from the long bone [24]. In another study, orofacial human bone marrow stromal cells proliferated more rapidly in vitro, indicating orofacial marrow stromal cells as a unique cell population [26]. Also, in comparison to the maxilla, the mandible demonstrated a higher bone remodeling dynamic in animal experiments with skeletally mature dogs [27].

Generally, the mandible is assumed to present higher amounts of collagen compared to the long bone, but posttranslational modifications of collagen, such as intermolecular crosslinking and lysine hydroxylation, are less mature in the mandible, which is meant to allow more flexibility and better resistance to constant exercise [23]. Bone mineral density (BMD) as a measure of bone strength is suggested to be higher in the body and symphysis area of the mandible compared to the spine and hip [28].

However, there are also studies showing the opposite: Rothweiler et al. compared the three-dimensional microstructure of alveolar and iliac bone and found a greater distance from mineralized tissue to the closest pore-vessel boundary in alveolar bone, which was associated with a worse regenerative potential of the alveolar bone [29]. Also, the more rapid proliferation of orofacial human bone marrow stromal cells in vitro could not be proven in vivo, where iliac crest cells formed more compacted bone and were more responsive to osteogenic induction [26]. This demonstrates that the specific properties of the mandible are still a matter of discussion. Even though there might be advantages in comparison with other bones, the influence of these distinct properties on long-term osseous healing seems to be neglectable.

In the present study, the preliminary histological observation revealed similar microstructures of the intersegmental bones of both interframes, although the fibula–fibula intersegmental bone seems to maintain the flap’s native morphological characteristics. Considering the macroscopic differences in shape and size between the mandibular and fibular bone, the preliminary morphological differences in osseous union between the intersegmental bones, as observed in the present study, indicate that the morphological differences can be devalued compared to the influence of mechanics or radiotherapy.

Several limitations account for these findings. Pseudarthrosis was only evaluated radiologically and the biomechanics of the intersegmental callus remain unknown. Due to strict inclusion criteria, the number of patients included is relatively small. Following our in-house standard, not all patients received two consecutive CBCTs because most patients suffered from malign tumors and routine tumor staging usually included a CT as staging, which limited the number of patients. Furthermore, there are varying time intervals between CBCT imaging and the retrospective study design. And although the methodology allowed a reduction of the impact of artefacts, interpretation may still be confounded by metal artefacts. These factors might also explain the insignificant differences in osseous union between superior and inferior parts of the gaps, although the inferior parts receive more axial load, which is beneficial for osseous healing [6].

## Conclusions

The results of the present study indicate that the bone interface at the intersegmental gap in mandibular reconstruction does not relevantly influence long-term bone healing. Reconstructive factors influencing mechanics or clinical factors like adjuvant radiotherapy seem to be more relevant factors in the development of pseudarthrosis. Further research should also focus on the biomechanical integrity of the callus over time in order to evaluate the reliability of radiological assessments.

## Data Availability

The datasets used and/or analysed during the current study are available from the corresponding author on reasonable request.

## Abbreviations

FFF: Fibula free flap
IOM: Interfragmentary movements
CBCT: Cone-beam computed tomography
TV: Total volume
HE: Hematoxylin and eosin
µCT: Micro-computed tomography
FE: Finite element
ARONJ: Antiresorptive agent-related osteonecrosis of the jaw
NCM: Neural crest mesenchyme
BMD: Bone mineral density
